# Comparative Study: Biguanide-, Sulfonamide-, and Natural Agent-Based Interventions in an In Vivo Experimental Diabetes Model

**DOI:** 10.3390/medicina61071151

**Published:** 2025-06-26

**Authors:** Iulian Tătaru, Ioannis Gardikiotis, Carmen Lidia Chiţescu, Oana-Maria Dragostin, Maria Dragan, Cerasela Gîrd, Alexandra-Simona Zamfir, Simona Iacob (Ciobotaru), Rodica Vatcu, Catalina Daniela Stan, Carmen Lăcrămioara Zamfir

**Affiliations:** 1 Department of Morphofunctional Sciences I, “Grigore T. Popa” University of Medicine and Pharmacy, 700115 Iași, Romania; iulian-alexandru-i-tataru@d.umfiasi.ro (I.T.); carmen.zamfir@umfiasi.ro (C.L.Z.); 2 CEMEX—Advanced Center for Research and Development in Experimental Medicine, “Grigore T. Popa” University of Medicine and Pharmacy, 700454 Iași, Romania; dr.gardikiotis@yahoo.com; 3 Department of Pharmaceutical Sciences, Research Centre in the Medical-Pharmaceutical Field, Faculty of Medicine and Pharmacy, “Dunarea de Jos” University of Galati, 800201 Galati, Romania; oana.dragostin@ugal.ro (O.-M.D.); simona.ciobotaru98@yahoo.com (S.I.); rodica.vatcu@gmail.com (R.V.); 4 Department of Pharmaceutical Sciences II, Faculty of Pharmacy, “Grigore T. Popa” University of Medicine and Pharmacy, 16 Universității Street, 700115 Iași, Romania; catalina.stan@umfiasi.ro; 5 Department of Pharmacognosy, Phytochemistry and Phytotherapy, Faculty of Pharmacy, University of Medicine and Pharmacy “Carol Davila”, 050474 Bucharest, Romania; cerasela.gird@umfcd.ro; 6 Department of Medical Sciences III, “Grigore T. Popa” University of Medicine and Pharmacy, 16 Universității Street, 700115 Iași, Romania; simona-zamfir@umfiasi.ro

**Keywords:** experimental diabetes, phytotherapy, hypoglycemic effect, pharmacokinetic parameters

## Abstract

*Background/Objectives:* In the context of diabetes, a multifactorial metabolic disorder with significant clinical implications, the present study investigates the hypoglycemic effects of a synthetic sulfonamide (S) administered individually and in combination with *Salvia officinalis* extract, compared to metformin as a standard therapeutic agent. *Methods*: An in vivo model of experimentally induced diabetes using alloxan was applied to Wistar female rats, divided into six experimental groups, including a healthy control group and a diabetes-induced, untreated group. Plasma concentrations of metformin and sulfonamide were quantified by high-performance liquid chromatography. The plasma steady-state concentrations of the pharmaceutical agents and their correlation with hypoglycemic effect were evaluated. *Results*: The combination of the synthetic sulfonamide (S) with *Salvia officinalis* extract resulted in the greatest reduction in blood glucose level (average value of 50.2%) compared to S (40.6%) or metformin (36.4%). All treatments demonstrated statistically significant differences in blood glucose levels compared to the diabetes-induced untreated group (*p* < 0.05). Pharmacokinetic analysis revealed a larger volume of distribution for the synthetic sulfonamide S (23.92 ± 8.40 L) compared to metformin (16.07 ± 5.60 L), consistent with its physicochemical properties. No significant correlation was found between plasma drug levels and glycemic response (*p* > 0.05). *Conclusions*: Our findings support the potential of combining standard therapeutic agents with natural alternatives such as *Salvia officinalis* to achieve improved glycemic control through complementary mechanisms. To the best of our knowledge, this is the first in vivo study to evaluate the combined effects of a sulfonylurea-type compound and *Salvia officinalis* extract in a diabetic animal model.

## 1. Introduction

Diabetes is a progressive, chronic disease that leads to severe complications with significant medical, social, and economic implications in the absence of proper management [1,2]. Diabetes causes anatomical, structural, and functional alterations within the vascular system that progressively impair multiple organs, ultimately resulting in multiorgan dysfunction [3]. Emerging evidence indicates that sustained dysregulation of glucose metabolism may also lead to nonvascular damage in organs such as the liver and heart and increase the risk of different types of cancer [4].

The concept of glucose toxicity (glucotoxicity) has been defined to describe the harmful effects of chronic hyperglycemia on cellular function, particularly in pancreatic β-cells, but also in other tissues and organs [5]. Chronic hyperglycemia plays a central role in the development of various diabetic complications by promoting a range of metabolic and structural alterations, such as activation of the polyol pathway, increased flux through the hexosamine biosynthetic pathway, increased synthesis of advanced glycation end-products (AGEs), overproduction of reactive oxygen species (ROS), dysregulation of hemodynamic control pathways, and activation of protein kinase C, which amplifies chronic inflammation by stimulating pro-inflammatory signaling pathways and enhancing cytokine expression (e.g., TNF-α, IL-1β) [4,6,7,8,9].

The molecular mechanisms through which diabetes exerts its effects on the body are diverse and include oxidative stress [10]; mitochondrial dysfunction [11]; elevated pro-inflammatory cytokines [12]; nitric oxide (NO) deficiency [13]; accumulation of AGEs, which trigger cellular damage and endothelial dysfunction [14]; and stiffening of the extracellular matrix [8].

Given the profound impact of hyperglycemia on cellular metabolism and mitochondrial function, maintaining optimal glycemic control is essential for preventing the progression of diabetes and its associated complications [2].

The present manuscript investigates the relationship between plasma concentrations of metformin and a synthetic compound with sulfonamide structure (S), alone or in combination with *Salvia officinalis* extract, and the corresponding blood glucose (BG) levels in alloxan-induced diabetic rats, as part of an in vivo experimental study. High-performance liquid chromatography (HPLC) was used for the analysis of the active substances’ plasma concentration.

Given the multifactorial pathogenesis of diabetes, involving both pancreatic β-cell dysfunction and peripheral insulin resistance, the therapeutic strategy in this study was to combine agents acting through complementary mechanisms. The sulphonamides exhibit insulinotropic activity and potential α-glucosidase inhibition, as supported by recent synthetic derivative studies [15] The S compound (substituted p-toluenesulfonamide) mechanism of action is presumed to partially mimic that of sulfonylureas, enhancing pancreatic insulin secretion and modulating peripheral glucose uptake, while *Salvia officinalis* extract, rich in bioactive polyphenols, may exerts insulin-sensitizing, antioxidant, and anti-inflammatory effects [16]. Recent molecular docking analyses have shown that these natural compounds interact with multiple molecular targets relevant to type 2 diabetes pathophysiology, including nuclear receptors, digestive and metabolic enzymes, and inflammatory transcription factors [17]. These interactions support the hypothesis that the sage bioactive compounds may modulate insulin signaling, oxidative stress, and inflammatory pathways concurrently. The rationale for combining these agents arises from the hypothesis that the co-administration may produce a complementary or synergistic glycemic effect by concurrently targeting insulin secretion, insulin sensitivity, and oxidative stress modulation. This integrated approach aligns with the growing interest in combined treatments that address multiple metabolic disruptions [18].

Moreover, to our knowledge, no in vivo studies have specifically addressed the combined administration of sulfonylurea-type compounds and natural extracts in animal models of diabetes.

The primary objective of the present paper was to compare the hypoglycemic effects of the tested interventions across experimental groups, including a healthy control group, an alloxan-induced untreated group, and treated groups, to determine whether the co-administration of Salvia officinalis extract could enhance the therapeutic effects of the synthetic compound. The secondary objective was to evaluate the plasma steady-state concentrations of metformin and compound S using high-performance liquid chromatography (HPLC), and to explore potential correlations between drug exposure and glycemic response.

This investigation is part of a broader in vivo experimental study on the impact of diabetes on the female reproductive system [19]. As a part of our previous work, the present manuscript focused on evaluating the glycemic response in relation to the pharmacokinetic aspects of the tested compounds.

## 2. Materials and Methods

### 2.1. Chemicals and Reagents

**Figure 1:**
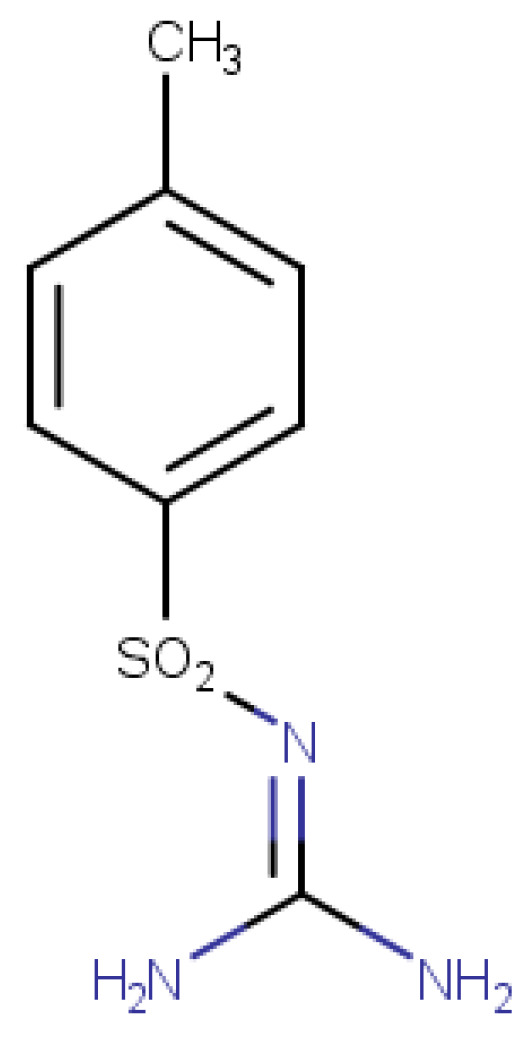
Structure of synthetic sulfonamide N-(diaminomethylene)-4-methylbenzenesulfonamide (C_8_H_11_O_2_N_3_S) [19].

Analytical standards for metformin and alloxan were provided by Alfa Aesar (Thermo Fisher Scientific, Waltham, MA, USA). The synthetic sulfonamide compound (Figure 1) was synthesized in-house via chemical synthesis starting from p-toluenesulfonamide (Sigma-Aldrich, ≥98%), urea (99.98%), potassium carbonate (K_2_CO_3_, 98%), and acetone (99.9%). The reaction was carried out under reflux conditions using a K/KM-5 reflux system (MRC- Laboratory Equipment, Holon, Israel). Although not a novel molecule, the sulfonamide used in this study serves as the core structure for a series of newly developed sulfonamide derivatives with potential antidiabetic activity, currently under investigation. Thus, the compound was evaluated for its intrinsic antidiabetic potential, as a preliminary step within this broader research context.

Structural confirmation of the synthesized sulfonamide compound was achieved by high-resolution mass spectrometry (HRMS) using the Vanquish Flex UHPLC System coupled with an Orbitrap Exploris 120 mass spectrometer (Thermo Fisher Scientific), operated in negative ionization mode. The selected ion had an exact mass of m/z 212.04992 [M–H]^−^, confirming the expected molecular structure. The full characterization protocol, including synthesis route and analytical confirmation by HRMS, is detailed in a previously published article by our group [19], and is referenced here to avoid redundancy in the current manuscript. The purity of this compound was established by determining its melting point, according to the literature [20], this being a fixed value and not a range (129 °C).

The plant material was purchased as a single-ingredient medicinal tea from certified pharmaceutical retailers in Romania. *Salvia officinalis* extract was obtained by reflux extraction using 50% ethanol, with a plant material-to-solvent ratio of 1:10 (g/mL), followed by lyophilization for concentration and preservation. The used extract was previously characterized for its chemical composition and antioxidant activity. The high-resolution mass spectrometry (HRMS) profiling revealed a complex matrix rich in bioactive polyphenols, including rosmarinic acid, salvianolic acid B, quercetin, kaempferol, and chlorogenic acid. In vitro antioxidant activity was demonstrated using standard assays, confirming its potent radical-scavenging and reducing properties. These results were detailed in the previously published study [18].

As part of the in vivo study setup, alloxan was formulated as an injectable solution in physiological saline at a concentration of 20 mg/mL. Metformin, the synthetic sulfonamide compound (S), and the sage extract were formulated as oral suspensions in 1% caboxymethylcellulose (CMC), at concentrations of 25 mg/mL for metformin and 75 mg/mL for both S and sage extract.

### 2.2. In Vivo Experimental Design

The in vivo experiment was conducted using Wistar female rats, obtained through the “Grigore T. Popa” University of Medicine and Pharmacy (UMF) Iași, from the Cantacuzino Institute in Bucharest, Romania. Throughout the experimental period, the animals were housed at the CEMEX biobase, within the UMF “Grigore T. Popa” Iași. All experimental procedures strictly adhered to international ethical regulations and were approved by the Medical Ethics Committee of UMF Iași (approval no. 169/22 March 2022). Additionally, the study followed the Guidelines on the Care and Use of Animals for Scientific Purposes (National Advisory Committee for Laboratory Animal Research, 2004 [21]), and was in accordance with national and international regulations (Romanian Law no. 206/27 May 2004 [22] and Directive EU/2010/63 on the protection of animals used for scientific purposes [23]). Female rats were selected in alignment with the broader research objective focused on diabetes-induced alterations of the female reproductive system.

Standard laboratory conditions were maintained throughout the experiment, including optimal temperature, natural light cycle, ventilation, hygiene, and access to a balanced nutritional regimen (food and water ad libitum). Direct contact with other animal species was strictly avoided.

The animals were randomly allocated into six experimental groups (seven animals per group according to the opinion of the ethics committee, and correlated with data from the literature), as follows, according to diabetic status and the type of treatment administered:

- Group 1 (L1)—healthy controls;
- Group 2 (L2)—diabetes-induced untreated controls;
- Group 3 (L3)—diabetes-induced, treated with metformin (300 mg/kg body weight/day);
- Group 4 (L4)—diabetes-induced, treated with the synthetic sulfonamide (S) (150 mg/kg body weight/day);
- Group 5 (L5)—diabetes-induced, treated with sage extract (150 mg/kg body weight/day each);
- Group 6 (L6)—diabetes-induced, treated with S and sage extract (150 mg/kg body weight/day each).

The inclusion criteria used were as follows: age (10–12 weeks old), sex (female), and health status. The exclusion criteria were health status and complications that occurred during the experiment.

Following a 7-day acclimatization period, diabetes was induced in groups 2–6 by a single intraperitoneal injection of alloxan at a dose of 150 mg/kg body weight. The dose of alloxan was established based on data reported in the scientific literature, which indicates that in rats, effective diabetogenic doses typically range from 40 to 200 mg/kg [24].

Blood glucose (BG) was assessed 5 days post-administration using peripheral tail vein blood. A threshold of 200 mg/dL was used to confirm severe hyperglycemia. As approximately 30% of the animals failed to reach this threshold after the first dose, a second identical dose of alloxan was administered at the beginning of the third week. BG level was re-evaluated after 5 days, confirming successful induction of diabetes in all animals from groups 2–6. The end of week 3 thus marked the point of confirmed diabetes and the onset of pharmacological interventions. Treatments with metformin, the synthetic sulfonamide compound (S), and sage extract were administered orally via gavage beginning at week 4 and continued daily for a duration of 5 weeks, using the doses specified above. The full experimental timeline extended over 8 weeks. The treatment duration was selected based on studies reporting effective durations of 2–8 weeks in chemically induced diabetic rodent models for assessing antidiabetic interventions [24,25,26].

The oral doses of metformin in rats reported in previous literature included values between 50 to 300 mg/kg [27,28]. The dose of compound S administered to the animals was established below 1/10 of the median lethal dose (LD_50_), based on a reported LD_50_ value of 2000 mg/kg body weight, in line with safety margins typically applied in subacute toxicity studies [29]. The dose of 150 mg/kg of *Salvia officinalis* extract was selected based on prior studies demonstrating antidiabetic efficacy and safety at comparable doses in chemically induced diabetic animal models [16,26,30].

To minimize potential confounders, all experimental procedures—including compound administration and blood glucose level measurements— were performed at the same time of day for all animals. Blood glucose (BG) levels were monitored weekly using a portable glucometer (tail vein blood).

At the end of the experimental period, blood samples were collected from the retro-orbital plexus under ketamine anesthesia (75 mg/kg body weight). Samples were collected into EDTA-coated tubes and stored at −20 °C until biochemical analyses were performed. Finally, the animals were humanely euthanized using Thiopental 0.01%.

### 2.3. Determination of Plasma Concentrations of Metformin and Compound S by High-Performance Liquid Chromatography (HPLC)

Extraction of the target compounds from whole blood samples was performed using a liquid–liquid extraction method, with dichloromethane as the organic solvent (sample: solvent ratio 1:2). The extraction was carried out under acidic conditions (pH 3, using 4% acetic acid). Following centrifugation, the organic phase was evaporated under a stream of high-purity nitrogen gas at 40 °C (Thermo Scientific, Bremen, Germany). The resulting dry residue was reconstituted in 500 µL of 10% methanol in water.

HPLC analysis was performed using an Agilent 1260 Infinity HPLC-DAD system (Agilent Technologies, Santa Clara, CA, USA). Separation was achieved on a 150-4.6 LiChrospher^®^RP-18, 5 µm, column (Merck, Darmstadt, Germany). The mobile phase consisted of phosphate buffer at pH 2.3 and acetonitrile, mixed in a ratio of 37:63 (*v*/*v*). The flow rate was set to 1.0 mL/min, and the total run time for each analysis was 30 min.

Calibration curves were constructed using standard solutions of metformin and the synthetic compound (S), prepared in methanol at 1 mg/mL and diluted with appropriate volumes of mobile phase to yield concentrations ranging from 5 to 25 µg/mL.

The physicochemical properties of metformin and compound S, including log P and pKa values, were calculated using MarvinSketch version 19.4 (ChemAxon, Budapest, Hungary).

### 2.4. Statistical Methods

Statistical analysis was performed using Microsoft Excel and XLSTAT 2023.3.0 (Addinsoft, Paris, France). Normality of data distribution was assessed using the Shapiro–Wilk test, while Levene’s test was applied to assess the homogeneity of variances across groups. When the condition for parametric testing was met (*p* > 0.05), one-way ANOVA was used to compare blood glucose levels between experimental groups at weeks 4 and 8. Tukey’s HSD and Dunnett’s post-hoc tests were used for multiple comparisons. Nonparametric Kruskal–Wallis test and Dunn’s post-hoc pairwise comparisons were applied if the normality test was not met (*p* < 0.050). To explore multivariate patterns and emphasize differences between groups, a principal component analysis (PCA) was performed. Correlation between plasma concentrations and glycemic response was analyzed using Pearson’s correlation coefficient (r). Where non-linear trends were observed, we fitted quadratic regression models to assess potential curvilinear relationships.

In order to assess potential complementary or synergistic effects of the combined treatment (S + sage) on glycemic control, a two-way ANOVA was performed, including the main effects of each compound and their interaction term.

A *p*-value < 0.05 was considered statistically significant throughout all analyses.

## 3. Results

### 3.1. Effect on Blood Glucose (BG) Level

BG levels measured in the treated groups both prior to and following treatment intervention exhibited a broad range of values, with considerable intra-group variability, reflecting a substantial degree of interindividual variation in glycemic response to the administered treatments. The control group (L1) maintained normoglycemic values throughout the experiment (97–116 mg/dL), whereas the untreated diabetic group (L2) exhibited the highest BG levels (390–413 mg/dL), confirming the development of severe hyperglycemia.

In the metformin-treated group, blood glucose (BG) values ranged from 72 to 522 mg/dL, with a mean of 224.8 ± 192.78 mg/dL. Within this group, BG showed considerable variability, possibly due to heterogeneous individual responses to the drug.

In comparison, the group treated with the synthetic sulfonamide compound (L4) exhibited moderate variability of BG level, ranging from 112 to 364 mg/dL, with a mean of 236.0 ± 94.70 mg/dL. Group 5, treated with sage, exhibited BG levels ranging from 232 mg/dL to 543 mg/dL, with a mean value of 358.6 ± 114.3 mg/dL. The group receiving both S and sage extract (L6) displayed a more homogeneous response, with the lowest BG levels between 98 and 224 mg/dL, and a mean of 139.2 ± 49.40 mg/dL.

**Figure 2:**
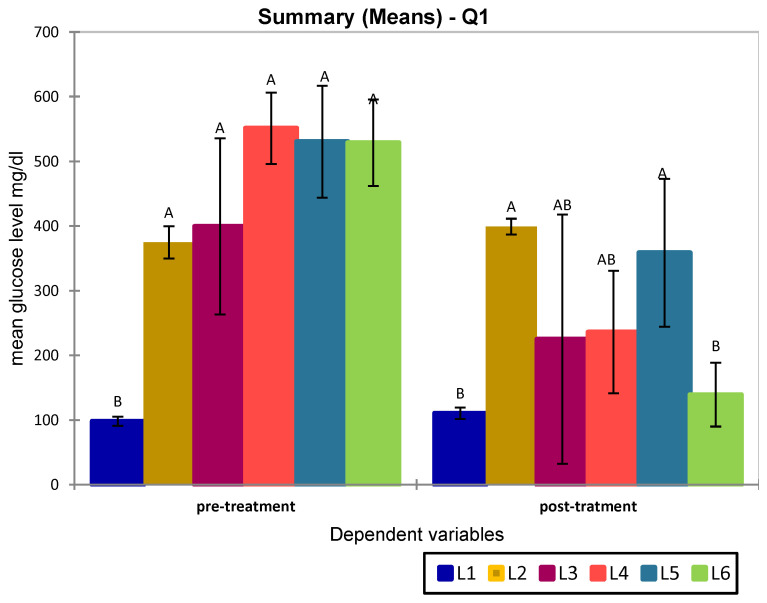
ANOVA of blood glucose values measured across the experimental groups (L1–L6) at baseline (end of week 3, pre-treatment) and after intervention (end of week 8, post-treatment). Letters above the bars indicate statistically significant differences between groups according to Tukey’s post-hoc test. Groups that share at least one common letter (e.g., A and AB or B and AB) are not significantly different, whereas those with no letters in common are significantly different (*p* < 0.05).

The distribution of BG values both prior to and after treatment met the assumptions of normality, according to the Shapiro–Wilk test (*p* > 0.05), thus allowing for parametric analysis. ANOVA analysis followed by post-hoc tests (Dunnett and Tukey HSD) revealed statistically significant differences at the end of week 3 (pre-treatment) between the non-diabetic control group (L1) and all other groups (R^2^ = 0.822; *p* < 0.05), which confirms the successful induction of diabetes (Figure 2). At week 8 (post-treatment), a reduction in blood glucose levels was observed in all treated groups. The one-way ANOVA model indicated that the differences between groups were statistically significant (R^2^ = 0.522, *p* = 0.006), supporting the treatment-related effects.

Dunnett’s post-hoc analysis (using the non-diabetic group L1 as reference) revealed that only the untreated diabetic group (L2) and the group treated with sage extract alone (L5) exhibited significantly elevated blood glucose levels compared to L1 (*p* = 0.010).

In contrast, no statistically significant differences were observed between L1 and the groups treated with metformin (L3), the S-only group (L4), or the combination of S + sage (L6), (*p* = 0.398, *p* = 0.317, and *p* = 0.992, respectively), suggesting a normalization of blood glucose level in these treated groups.

Applying Dunnett’s post-hoc analysis by using the diabetic untreated group L2 as reference control, statistically significant differences were found for both the healthy control group (L1) (*p* = 0.009) and group L6, treated with S and sage extract (*p* = 0.014). Although groups L3 and L4 (sulphonamide and metformin monotherapies) showed blood reductions in blood glucose levels, they did not reach statistical significance when compared to the untreated diabetic group (L2) (L2 vs. L3, *p* = 0.130; L2 vs. L4, *p* = 0.167). This trend is reflected in the ANOVA output, where L3 and L4 shared overlapping letters (A, B) with L2, indicating a lack of significant distinction at the 95% confidence level (Figure 2). The group treated with sage extract L5 showed the lowest glucose reduction (L2 vs. L5, *p* = 0.972).

To quantify the hypoglycemic effect of the applied treatments, the percentage decrease in blood glucose was calculated based on pre- and post-treatment values using the following formula:

$$Absolute\;decrease\;in\;blood\;glucose\;\%=\frac{blood\;glucose\;(pre-treatment)-blood\;glucose\;(post-treatment)}{blood\;glucose\;(pre-treatment)}\times 100$$

The obtained values, expressed as mean ± standard deviation, indicate a potent hypoglycemic effect across all treated groups.

**Figure 3:**
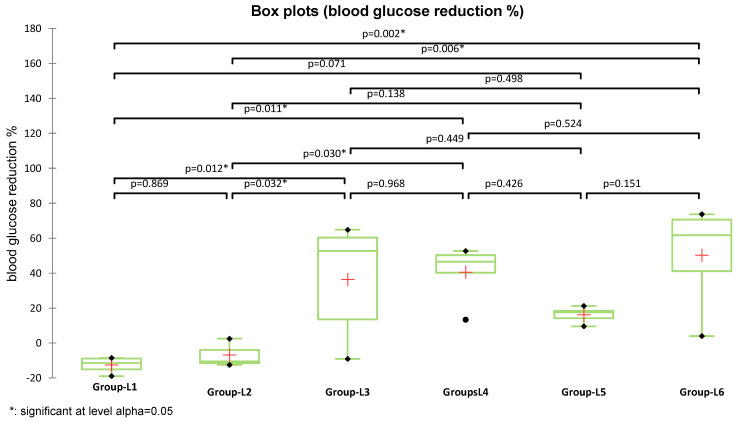
Box plots representing the percentage reduction in blood glucose levels for experimental groups (L1–L6). The red crosses represent group means; horizontal lines within the boxes indicate medians. The horizontal line within each box indicates the median, while the red cross (“+”) denotes the group mean.

The average blood glucose reduction (%) was higher in groups treated with S (L4) (40.95 ± 15.94%) and with S and sage (L6) (50.22 ± 28.2%), compared to the group treated with metformin (L3) (36.46 ± 32.5%). As the assumption of normality was not met across all experimental groups, differences in blood glucose reduction were analyzed using the nonparametric Kruskal–Wallis test, which revealed a statistically significant overall effect (K = 15.397, *p* = 0.009). Subsequent Dunn’s post-hoc pairwise comparisons identified significant differences between groups (Figure 3). Both metformin (used in L3) and the S compound (used in L4) induced statistically significant glycemic improvements compared to the diabetes-induced untreated group (L2), with *p* = 0.032 and *p* = 0.030, respectively. Moreover, the combination treatment (L6) demonstrated the greatest BG reduction and a significant difference versus L2 (*p* = 0.006), the group treated with sage extract alone, supporting its enhanced therapeutic potential. Although the reduction observed in the sage-only group (L5) reached 16.16% compared to L2, the difference was not statistically significant (*p* = 0.138).

To further explore potential synergistic interactions between the treatments, two-way ANOVA models—including the main effects (S and sage) and their interaction term (S + sage)—and logistic regression analyses were applied to blood glucose response categories in groups L4, L5, and L6. The overall model was not statistically significant (F(2,12) = 2.337, *p* = 0.139), and the interaction itself showed no significant contribution to explaining variance (*p* = 1.000). These results showed the absence of a synergistic effect; the marked glycemic response observed in group L6, treated with S and sage, likely reflects an additive effect under the tested conditions.

### 3.2. Quantification and Evaluation of Plasma Level of Metformin and the Synthetic Compound (S)

High-performance liquid chromatography (HPLC) enabled the accurate quantification of both metformin and the synthetic compound (S) in plasma samples. The calibration curves for both metformin and the synthetic sulfonamide compound (S) demonstrated strong linearity within the tested concentration range (2.5–25 μg/mL), with correlation coefficients (R^2^) of 0.9989 and 0.9986, respectively. The limits of detection (LOD) and quantification (LOQ) were calculated using the calibration curve method, based on the standard deviation of the intercept and the slope (LOD = 3σ/S; LOQ = 10σ/S). The LOD and LOQ values were 1.055 μg/mL and 3.199 μg/mL for metformin, and 1.060 μg/mL and 3.213 μg/mL for compound S, respectively. No endogenous compounds were found to interfere with the analyte or internal standard peaks.

**Figure 4:**
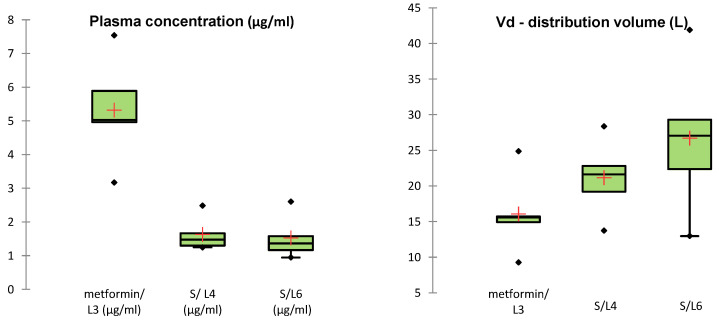
Pharmacokinetic parameters of metformin and the synthetic compound in experimental groups.

Quantitative analysis showed an average plasma concentration of metformin of 5.32 ± 1.58 μg/mL, ranging from 3.173 μg/mL to 7.54 μg/mL. For the synthetic compound S, the average plasma concentration was 1.58 ± 0.54 μg/mL, ranging between 0.94 μg/mL and 2.60 μg/mL (Figure 4). The plasma concentration distribution for both compounds was consistent with a normal distribution (*p* > 0.05, Shapiro–Wilk test).

The volume of distribution at steady state (*Vd*) was calculated using the following formula:

$$Vd=\frac{D}{Css}(L)$$

where *D* represents the administered dose, calculated individually based on each animal’s body weight (mg/kg), and *Css* is the steady-state plasma concentration (mg/L).

The results revealed a lower volume of distribution for metformin (16.07 ± 5.60 L) compared to the synthetic compound S (23.92 ± 8.40 L). Moreover, differences were observed between the distribution volumes of compound S in groups L4 and L6, suggesting differences in pharmacokinetic profiles (Figure 4).

**Figure 5:**
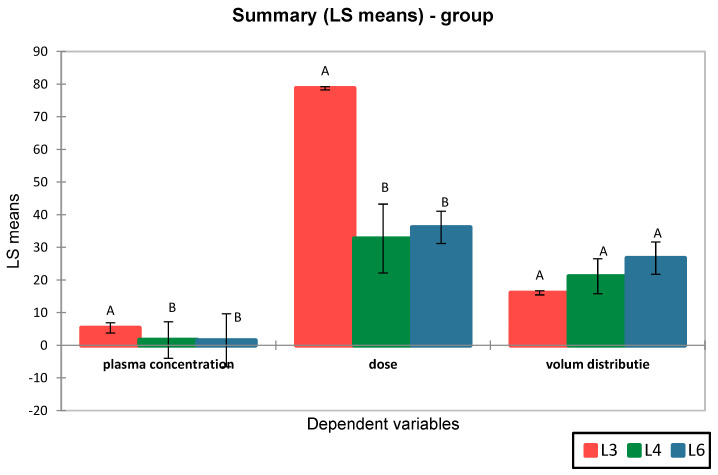
Comparison of pharmacokinetic parameters across groups L3 (metformin), L4 (synthetic compound), and L6 (compound + sage extract). Least squares means are shown for plasma concentration (μg/mL), administered dose (mg/kg), and volume of distribution (L). Error bars represent standard deviation. Different letters (A, B) indicate statistically significant differences between groups for each dependent variable (*p* < 0.05, ANOVA with post-hoc test).

ANOVA analysis followed by Tukey’s post-hoc test revealed that although the mean Vd values differed between groups, the *p*-values were > 0.05, indicating that the observed differences were not statistically significant. These findings suggest that the volume of distribution of compound S is comparable between groups L4 and L6 and does not significantly differ from that of metformin (L3), indicating a generally similar systemic distribution pattern (Figure 5).

### 3.3. Correlation and Regression Analyses of Dose–Response Relationships

Linear regression analysis between the administered dose and plasma concentration showed a statistically significant positive correlation (R^2^ = 0.701, *p* < 0.0001), indicating dose-proportional absorption of the compounds.

The correlation between administered dose and blood glucose reduction was further assessed using Pearson’s coefficient. When analyzing individual treatment groups, a positive dose–response trend was observed in the metformin group (r = 0.805, *p* = 0.089), suggesting a dose-dependent hypoglycemic effect, although the result did not reach statistical significance. In the group treated with the synthetic compound (L4), no correlation was found (r = 0.33, *p* = 0.482), while in group L6, a moderate, but non-significant association was observed (r = 0.68, *p* = 0.073).

**Figure 6:**
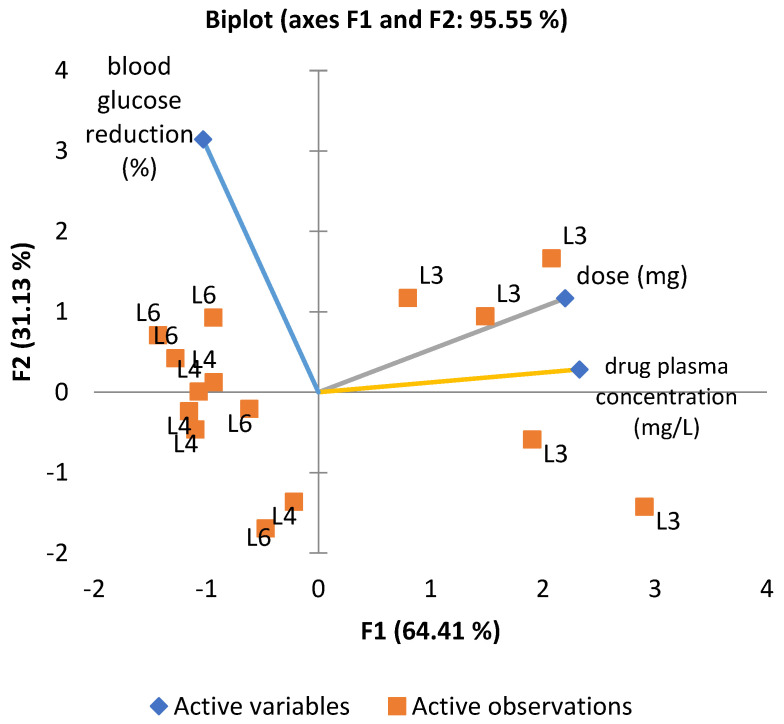
Biplot representation (PCA) illustrating the relationship between administered dose (mg), plasma concentration (mg/L), and blood glucose reduction (%) across the three experimental groups.

To explore the combined influence of administered dose, plasma concentration, and glucose-lowering effect, a principal component analysis (PCA) was performed (Figure 6). The first two components (F1 and F2) accounted for 95.55% of the total variance. The loading plot revealed a strong correlation between dose and plasma concentration vectors, whereas the vector for glucose reduction was oriented orthogonally, confirming the absence of a direct linear relationship. The positioning of groups L4 and L6 in the upper-left quadrant of the PCA biplot suggests similar profiles, distinct from group L3, treated with metformin. Although L6 shows a slightly more substantial association with blood glucose reduction, both L4 and L6 appear to cluster in relation to this effect.

**Figure 7:**
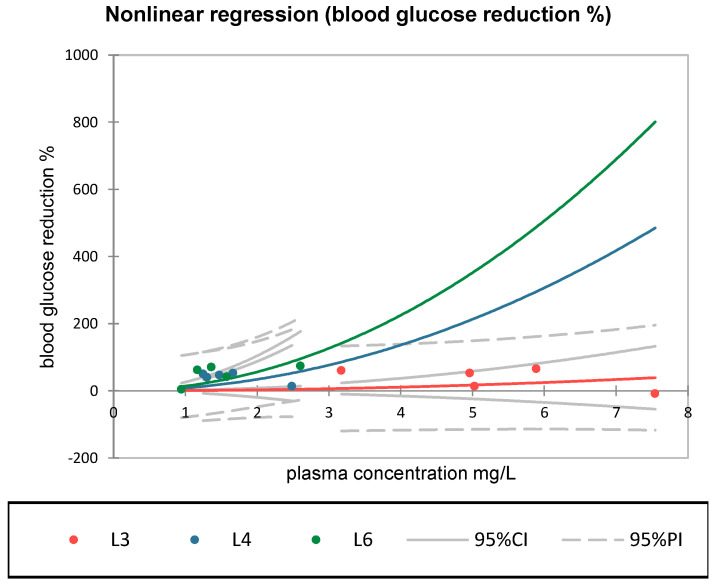
Nonlinear regression of the relationship between plasma concentration (mg/L) and blood glucose reduction (%) across the three experimental groups: L3—metformin (red), L4—synthetic compound (blue), L6—compound + sage (green). The fitted curves follow the equation *Y = pr*_1_ *×* (*X*)^2^, with 95% confidence intervals (CI) and prediction intervals (PI) displayed.

A multiple linear regression model on dose and plasma concentration influencing blood glucose reduction did not reveal a statistically significant relationship (R^2^ = 0.192, *p* = 0.277), which may reflect complex and non-linear pharmacodynamics that determine the hypoglycemic response of the tested compounds (Figure 7). In contrast, non-linear regression analysis highlighted relevant differences between groups. In group L6 (synthetic compound + sage), the exponential model was statistically significant (pr_1_ = 14.07, *p* < 0.05), indicating a potential activation of the hypoglycemic effect at moderate plasma concentrations of S compound (within the 1–2.5 mg/L range). This curvilinear relationship was not observed in groups L3 or L4, where the correlations were weak or statistically insignificant. The relevance of these curves lies in the comparative behavior within the observed range, where group L6 showed the most pronounced and steepest response, due to a non-linear effect. The steep increases observed at higher concentrations (e.g., >3 mg/L) are extrapolations of the fitted model and should be interpreted with caution.

## 4. Discussion

Within the present study, the efficacy in reducing blood glucose level of a synthetic sulfonamide (S) (administered in L4), *Salvia officinalis* extract alone (L5), and their combination (L6) was evaluated and compared to that of the standard antidiabetic agent metformin (used in L3). The potential synergistic or additive interactions resulting from the co-administration of sage extract and S were investigated. Systemic exposure to the active compound was assessed through plasma concentration analysis using HPLC.

The observed differences in the steady-state plasma concentration of compound S compared to metformin can be attributed to distinct physicochemical properties. Synthetic compound S displays higher lipophilicity (log P = 0.42) and a lower pKa (~5–6) (MarvinSketch version 19.4), which favors its presence in the unionized form at physiological pH, enhancing membrane permeability and resulting in broader tissue distribution [31]. The observations are consistent with data reported for other sulfonamide analogues with antidiabetic potential, which exhibit comparable pharmacokinetic characteristics attributed to partial lipophilicity and weak protein binding [32,33]. In addition, such compounds tend to maintain plasma stability and demonstrate efficient accumulation in target tissues, including the pancreas, as revealed by biodistribution studies using radiolabeled analogues [33].

Across all groups, Pearson’s correlation analysis revealed no statistically significant associations between dose and glycemic response, though a positive trend was noted for metformin (r = 0.805) and the S + sage combination (r = 0.68). A nonlinear regression analysis demonstrated varying degrees of concentration–response among the treated groups. Notably, only the combination group (L6) exhibited a statistically significant model (pr_1_ = 14.07, *p* < 0.05), consistent with a concentration-dependent amplification of the hypoglycemic response at moderate plasma levels.

In terms of hypoglycemic effect, the synthetic sulfonamide compound administered in L4 induced an average BG reduction of 40.6%, higher than the value observed for metformin (36.4%), associated with lower interindividual variability (SD = 15.9). Group L4 showed no significant nonlinear association between plasma concentration and blood glucose reduction, indicating limited concentration–response consistency. The result is consistent with clinical findings, which reported no significant correlation between plasma levels of tolbutamide (a classical sulfonylurea structurally related to compound S) and glycemic response in type 2 diabetic patients, despite a significant dose–concentration relationship [34].

Given the structural similarity to sulfonylureas, it is reasonable to expect a substantial glycemic response for the S compound, presuming a similar mechanism of stimulation involving insulin release from pancreatic β-cells [15]. This occurs by binding to the SUR1 (sulfonylurea receptor 1) subunit located on ATP-sensitive potassium (KATP) channels in the pancreatic β-cell membrane. This binding leads to the closure of KATP channels, preventing the efflux of potassium ions and resulting in membrane depolarization. The depolarization opens voltage-dependent calcium channels, allowing calcium influx and increasing intracellular calcium concentration. The elevated calcium level subsequently triggers the exocytosis of insulin-containing granules, thereby enhancing insulin secretion [35,36].

Moreover, recent studies on synthetic sulfonamide derivatives have demonstrated additional mechanisms—including α-glucosidase inhibition, delayed carbohydrate absorption, and increased cellular glucose uptake—that may contribute to their enhanced glucose-lowering effect [15,32,33]. Molecular docking studies suggest that sulfonamide moieties interact efficiently with key catalytic residues in the α-glucosidase active site, forming hydrogen bonds that mimic the transition state of the substrate [15].

This mechanism is distinct from that of metformin, which lowers blood glucose primarily by inhibiting hepatic gluconeogenesis via activation of AMP-activated protein kinase (AMPK) and mitochondrial complex I inhibition, while enhancing peripheral glucose uptake and reducing intestinal glucose absorption through local mucosal effects [37,38].

The literature is consistent with the observed hypoglycemic effect in group 4, which was treated exclusively with the synthetic sulfonamide compound.

As expected, metformin administration resulted in a moderate average BG reduction of 36.46%, consistent with previous findings reporting reductions between 25 and 35% after chronic treatment in both animal and clinical studies [37]. However, the variability in BG levels within this group was substantial (72–522 mg/dL; SD = 192.78), consistent with prior reports on the drug’s heterogeneous efficacy in diabetic animal models [27,39]. This variability may primarily arise from individual differences in absorption, distribution, and bioavailability of metformin, which are influenced by factors such as gastrointestinal transport, first-pass metabolism, and organic cation transporter activity [40,41,42].

The metformin plasma concentrations observed in group L3 are also consistent with previous reports in animal models. Chaudhari et al. (2020) reported steady-state metformin levels of 2.85 to 2.92 mg/L in mice treated with 200 mg/kg/day metformin in drinking water [39]. Considering the higher dose used in our study (300 mg/kg) and potential species-specific differences in absorption and distribution between mice and rats, the concentrations measured in our rat model are in agreement with concentrations reported to produce therapeutic effects in animal studies [39]. Values obtained for the distribution volumes are not uncommon for metformin, as the drug is known to exhibit extensive tissue distribution mediated primarily by organic cation transporters (OCTs), particularly OCT1 and OCT2 [41]. The literature reports Vd values for metformin in humans ranging from 63 to over 600 L following oral administration, due to the compound’s low plasma protein binding and high intracellular accumulation in the liver, kidneys, and other tissues [41,43].

No significant linear (R^2^ = 0.010, *p* = 0.722) or nonlinear (pr_1_ = 0.687, *p* > 0.05) correlation was observed between metformin plasma level and hypoglycemic response. This finding aligns with the known pharmacokinetic and pharmacodynamic complexity of metformin and supports the hypothesis that its glucose-lowering effect is not strictly dependent on systemic concentrations, but also involves local mechanisms [44].

As previously reported by Choi et al. (2006), approximately 65% of orally administered metformin in rats is subject to gastrointestinal and hepatic first-pass effects, resulting in limited systemic bioavailability [42]. The authors estimated that more than half of the administered drug exerts its effect locally within the gastrointestinal tract, suggesting that metformin’s glucose-lowering activity may be mediated by local, pre-systemic mechanisms [42].

The lack of a correlation between metformin plasma levels and glycemic response in group L3 is consistent to current pharmacological evidence indicating that metformin exerts its antihyperglycemic effect primarily through local actions in the intestinal mucosa—such as AMPK activation and modulation of glucose transporters—as well as hepatic mechanisms including inhibition of gluconeogenesis and regulation of the gut–brain–liver axis, rather than via concentration-dependent systemic effects [42,45].

Despite the relatively low range of plasma concentrations of S compound measured in group 6, treated with the synthetic compound S combined with *Salvia officinalis* extract, this group exhibited the highest mean percentage reduction in blood glucose (50.22%) and low interindividual variability (SD = 28.8%), indicating a more consistent glycemic response compared to the metformin-treated group (L3).

*Salvia officinalis* exerts antioxidant and anti-inflammatory effects through multiple mechanisms, including direct free radical scavenging, inhibition of pro-inflammatory cytokine release (e.g., tumor necrosis factor-alpha (TNF-α), interleukin-6 (IL-6)), and modulation of redox-sensitive transcription factors such as nuclear factor kappa B (NF-κB) and nuclear factor erythroid 2-related factor 2 (Nrf2) [46].

It has also been identified as a possible antidiabetic agent, with demonstrated activity in vitro [25], in vivo [16,47,48], and in randomized controlled trials [49]. The modulation of glucose metabolism by *Salvia officinalis* has been linked to delayed carbohydrate absorption and improved peripheral insulin sensitivity [18,50,51].

Recent network pharmacology and molecular docking studies have shown that various molecular targets of current antidiabetic drugs are likely modulated by Salvia officinalis compounds. Salvianolic acid B and rosmarinic acid, in particular, have shown strong binding affinities in silico towards key proteins like peroxisome proliferator-activated receptor gamma (PPARγ), glycogen synthase kinase 3 beta (GSK3B), subunit of nuclear factor (NF-κB), DPP4 (dipeptidyl peptidase-4), RELA (NF-κB p65 subunit), and amylase alpha 1A (AMY1A) [17].

An in vitro and in vivo study established the mechanism of action of an important bioactive component in sage extract. Salvianolic acid A exerts its antidiabetic effects primarily by improving mitochondrial function, enhancing ATP production, and reducing mitochondrial membrane potential, which contributes to decreased oxidative stress. Furthermore, it increases glucose consumption and uptake in hepatic and muscle cells without stimulating insulin secretion or activating the PI3K/Akt pathway, instead acting via AMPK activation [25].

Several studies have demonstrated that other sage bioactive compounds, such as caffeic acid, chlorogenic acid, and apigenin, exerts hypoglycemic effects via multiple mechanisms, including enhancement of peripheral insulin sensitivity, reduction of hepatic gluconeogenesis, modulation of glucose uptake through GLUT-4 translocation, reduction of oxidative stress, and inhibition of inflammatory cascades involved in insulin resistance [50,52,53].

Recent in silico and in vivo studies also suggest that rosmarinic acid interacts with key molecular targets, supporting its potential role in inhibiting drug efflux transporters, modulating metabolic enzymes, and influencing incretin signaling, inflammation, and carbohydrate metabolism [51].

Moreover, studies have demonstrated dose-dependent antidiabetic effects of Salvia officinalis extract in streptozotocin-induced diabetic rats, with efficacy comparable to glibenclamide [15].

Linear correlation analysis (Pearson’s r = 0.595, *p* = 0.290) did not reveal a statistically significant association between S steady-state plasma concentration and glucose-lowering effect in group L6. Two-way ANOVA did not identify a statistically significant interaction between the sulfonamide compound (S) and *Salvia officinalis* extract (*p* = 1.000), suggesting the absence of a synergistic effect.

In contrast to groups L3 or L4, group L6 exhibited a statistically significant exponential concentration–response relationship (pr_1_ = 14.07, *p* < 0.05), with enhanced glycemic reduction at 1–2.5 mg/L. This pattern may reflect a complementary pharmacologic effect arising from *Salvia officinalis* co-administration. Our findings align with reported clinical evidence showing that natural products can improve glycemic control alongside standard medication in patients with diabetes, even in the absence of demonstrated synergy [54].

The present study supports the hypothesis that combining agents with complementary mechanisms—such as insulin-sensitizing, antioxidant, and anti-inflammatory pathways—can improve glycemic control and mitigate complications of diabetes.

### Limitations and Generalizability

Limitations of the study include the biological variability of glycemic responses in diabetic rats, single time-point blood glucose level assessments, and the sample size, which may have influenced the ability to detect statistically significant differences between treatment groups.

While the observed effects, especially the glycemic reduction in the sulfonamide + *Salvia officinalis* group, are promising, caution must be exercised in extrapolating these results directly to clinical settings.

Further studies based on a more comprehensive characterization of the metabolic response, including glucose tolerance tests (GTT), insulin tolerance tests (ITT), or insulinemia profiling would be necessary, to provide deeper insights into insulin sensitivity and β-cell function, and to strengthen the translational relevance of these findings.

## 5. Conclusions

This study compared hypoglycemic effects and the plasma steady-state concentrations of a synthetic sulfonamide compound (S), alone and in combination with *Salvia officinalis* extract, to those of metformin in an alloxan-induced diabetic rat model. Group L6, which received the combination of a synthetic sulfonamide and *Salvia officinalis* extract, showed the most pronounced reduction in blood glucose levels and consistency in response. The improved glycemic control observed in group L6 is most likely the result of a complementary pharmacologic effect arising from the co-administration of *Salvia officinalis* and compound S, as synergy was not demonstrated.

The HPLC method enabled accurate quantification of plasma drug levels and allowed the calculation of distribution volumes of the active substance, which appear to be influenced by their physicochemical properties. No direct linear relationship was found between systemic exposure and therapeutic effect. However, in group L6, nonlinear regression analysis revealed a statistically significant exponential concentration–effect relationship.

These findings highlight the complexity of dose–response relationships in antidiabetic therapy and underline the potential of phytochemical combination strategies to enhance therapeutic efficacy through complementary mechanisms. Further research is needed to investigate the mechanistic basis of the observed effects and to evaluate the long-term metabolic and safety profiles of such combinations in diabetic models.

## Data Availability

No new data were created.
